# Altered cortical thickness development in 22q11.2 deletion syndrome and association with psychotic symptoms

**DOI:** 10.1038/s41380-021-01209-8

**Published:** 2021-07-12

**Authors:** Joëlle Bagautdinova, Daniela Zöller, Marie Schaer, Maria Carmela Padula, Valentina Mancini, Maude Schneider, Stephan Eliez

**Affiliations:** 1 Developmental Imaging and Psychopathology Laboratory, Department of Psychiatry, Faculty of Medicine, University of Geneva, Geneva, Switzerland; 2 Medical Image Processing Laboratory, Institute of Bioengineering, École Polytechnique Fédérale de Lausanne (EPFL), Lausanne, Switzerland; 3 Department of Radiology and Medical Informatics, University of Geneva, Geneva, Switzerland; 4 Clinical Psychology Unit for Intellectual and Developmental Disabilities, Faculty of Psychology and Educational Sciences, University of Geneva, Geneva, Switzerland

**Keywords:** Schizophrenia, Neuroscience

## Abstract

Schizophrenia has been extensively associated with reduced cortical thickness (CT), and its neurodevelopmental origin is increasingly acknowledged. However, the exact timing and extent of alterations occurring in preclinical phases remain unclear. With a high prevalence of psychosis, 22q11.2 deletion syndrome (22q11DS) is a neurogenetic disorder that represents a unique opportunity to examine brain maturation in high-risk individuals. In this study, we quantified trajectories of CT maturation in 22q11DS and examined the association of CT development with the emergence of psychotic symptoms. Longitudinal structural MRI data with 1–6 time points were collected from 324 participants aged 5–35 years (*N* = 148 22q11DS, *N* = 176 controls), resulting in a total of 636 scans (*N* = 334 22q11DS, *N* = 302 controls). Mixed model regression analyses were used to compare CT trajectories between participants with 22q11DS and controls. Further, CT trajectories were compared between participants with 22q11DS who developed (*N* = 61, 146 scans), or remained exempt of (*N* = 47; 98 scans) positive psychotic symptoms during development. Compared to controls, participants with 22q11DS showed widespread increased CT, focal reductions in the posterior cingulate gyrus and superior temporal gyrus (STG), and accelerated cortical thinning during adolescence, mainly in frontotemporal regions. Within 22q11DS, individuals who developed psychotic symptoms showed exacerbated cortical thinning in the right STG. Together, these findings suggest that genetic predisposition for psychosis is associated with increased CT starting from childhood and altered maturational trajectories of CT during adolescence, affecting predominantly frontotemporal regions. In addition, accelerated thinning in the STG may represent an early biomarker associated with the emergence of psychotic symptoms.

## Introduction

In recent years, considerable research efforts in schizophrenia have provided extensive support for a neurodevelopmental origin of the illness involving altered gray matter structure. Studies consistently report reduced volume and cortical thickness (CT) in frontal, temporal, and cingulate areas [1–6], suggesting alterations in the neuropil of affected individuals. Post-mortem studies revealing decreased synaptic density in brain tissue of patients with schizophrenia provide additional supportive evidence (reviewed in [7]). Importantly, findings suggest that structural alterations appear in a progressive manner, with milder gray matter reductions in individuals at clinical high risk and gradually more aggravated differences in patients with first-episode psychosis and chronic schizophrenia [4, 8, 9].

While the neurodevelopmental origin of schizophrenia is therefore increasingly validated, gaining insights into the period that precedes the onset of the disorder remains challenging. Prospective studies on ultra-high-risk individuals have emerged to address this question and have shown steeper rates of cortical thinning in individuals who eventually convert to psychosis, affecting frontotemporal regions [9–11], as well as cingulate, parietal and insular areas [11]. However, studies investigating ultra-high-risk individuals have been limited by their number of follow-up scans (e.g., 1 follow-up) and did not assess neurodevelopment in at-risk individuals before the onset of psychotic symptoms.

With a 30–40% conversion rate to psychosis, 22q11.2 Deletion Syndrome (22q11DS) is considered a genetic model to study the developmental factors related to the emergence of psychosis [12, 13]. The syndrome is caused by a deletion of 1.5–3 Mb on the long arm of chromosome 22. Syndromic individuals are typically identified very early during development and can therefore be followed up starting from childhood, providing a unique opportunity to gain insight into the neurodevelopmental mechanisms preceding the onset of psychosis. On a neuroanatomical level, studies have generally reported widespread increased CT in 22q11DS and focal decreases in posterior cingulate and superior temporal gyri (STG) compared to healthy controls [14–16], a finding that was recently confirmed in a large multisite study [17]. However, the developmental trajectory of CT in 22q11DS remains unclear [18], as some reported faster thinning [19], while others found slower thinning [16, 20], a lack of thinning [15] or no difference in thinning [17] in 22q11DS compared to controls. A large-scale longitudinal study investigating gray matter development with a fine-grained temporal and topological resolution is thus needed to clarify neuroanatomical development in 22q11DS.

Evidence furthermore suggests that findings of progressive cortical thinning found across clinical stages of idiopathic schizophrenia may also be evident in 22q11DS. Studies comparing CT changes associated with the presence of psychotic symptoms in 22q11DS found steeper gray matter decline in frontal [21–23], temporal [21], cingulate [23], and parietal regions [23, 24]. However, these findings were limited by their sample size (*N* = 44–135) and follow-up assessments (typically 2–3 scans), preventing a precise delineation of CT alterations involved in the emergence of psychotic symptoms in 22q11DS.

Another potentially important, yet understudied component of brain structure is surface area (SA). Studies have shown reduced SA in 22q11DS [14, 16, 17] and idiopathic schizophrenia [25, 26], as well as steeper cross-sectional decline over age in 22q11DS [16]. No alterations were found when comparing individuals with 22q11DS with and without psychotic symptoms [15, 17].

Using the largest-ever longitudinal data set of individuals with 22q11DS and controls comprising 636 scans with up to six assessments covering an age span of 5–35 years, our first objective was to provide a robust characterization of developmental trajectories of CT and SA in 22q11DS and controls. Next, using a subset of 244 scans from individuals with 22q11DS with up to five assessments covering an age span of 6 to 28 years, our second objective was to confirm that the onset of positive psychotic symptoms during development is characterized by accelerated thinning trajectories. Mixed models regression was applied at each cortical vertex to accurately capture developmental trajectories with high topological precision.

## Method

### Participants

Participants were recruited in the context of an ongoing longitudinal study, through parent associations and word of mouth [19, 27]. Written informed consent was received from participants and their parents (for subjects <18 years old), under protocols approved by the cantonal research ethics commission. The presence of a 22q11.2 microdeletion was confirmed using quantitative fluorescent polymerase chain reaction (QF-PCR).

**Fig. 1:**
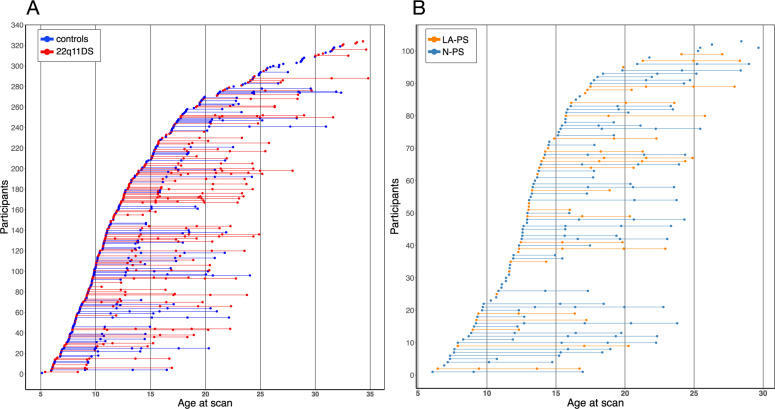
Scan distribution across the included age range. Scan distribution for the analyses comparing (**A**) 22q11DS and controls and (**B**) LA-PS vs N-PS subgroups within 22q11DS. For 22q11DS and control groups, the sample consisted of 324 participants (*N* = 148 22q11DS (73 male, 75 female); *N* = 176 controls (90 male, 86 female)) aged 5–35 years who contributed between 1 and 6 scans, resulting in a total of 636 scans (*N* = 334) 22q11DS (153 male, 181 female); *N* = 302 controls (143 male, 159 female). For LA-PS and N-PS groups, the sample consisted of 108 individuals with 22q11DS (*N* = 47 N-PS (24 male, 23 female); *N* = 61 LA-PS (32 male, 29 female)) aged 6–28 years who contributed between 1 and 5 scans, resulting in a total of 244 scans (*N* = 98) N-PS (48 male, 50 female); *N* = 146 LA-PS (68 male, 78 female).

For the modeling of CT development in 22q11DS and controls, the sample consisted of 324 participants (*N* = 148 22q11DS (50.7% female); *N* = 176 controls (58.1% female)) aged 5–35 years who contributed 1–6 scans, resulting in a total of 636 scans (*N* = 334 22q11DS; *N* = 302 controls) (Fig. 1A). Controls were carefully screened to exclude any neurological or psychiatric disorders.

Next, to investigate the association of CT maturation with the emergence of positive psychotic symptoms, we selected a subsample of participants with 22q11DS who underwent the structured interview for psychosis-risk syndromes (SIPS) [28]. Specifically, participants were classified as having “lifetime attenuated psychotic symptoms” (LA-PS) when they fulfilled the attenuated positive symptoms criterion of the ultra-high-risk status, defined as a score ≥3 at any of the positive symptoms subscales P1-P5 (P1: unusual thought content/delusional ideas, P2: suspiciousness/persecutory ideas, P3: grandiose ideas, P4: perceptual abnormalities/hallucinations, P5: disorganized communication) at any of their assessments. Otherwise, they were classified as having “no psychotic symptoms” (N-PS). The sample selected based on these criteria consisted of 108 participants with 22q11DS (*N* = 47 N-PS, 48.9% female; *N* = 61 LA-PS, 47.5% female) aged 6–28 years who contributed 1–5 scans, resulting in a total of 244 scans (*N* = 98 N-PS; *N* = 146 LA-PS) (Fig. 1B). In addition, to assess CT maturation in individuals with 22q11DS who develop overt psychosis, we conducted an exploratory analysis comparing three subgroups: individuals with low psychotic symptoms (N-PS, *N* = 47), individuals with psychotic symptoms who did not develop psychosis (LA-PS no psychosis, *N* = 49), and individuals with psychotic symptoms and a diagnosis of psychosis (LA-PS with psychosis, *N* = 12) (Fig. S1). The presence of psychosis was established when participants fulfilled the criteria for a diagnosis at any of their time points. Supplementary Method and Table S1 contain details on participant demographics, neurocognitive measures, and psychiatric assessment.

### Imaging

T1-weighted MRI anatomical brain scans were acquired using three different scanners: a 1.5T Philips Intera scanner (158 scans), a 3T Siemens Trio scanner (300 scans), and a 3T Siemens Prisma scanner (178 scans); see Figs. S2 and S3, Table S2. The proportion of scans acquired with each scanner did not differ between 22q11DS and controls (*p* = 0.07) or LA-PS and N-PS groups (*p* = 0.83).

Anatomical segmentation of T1-weighted images was performed using FreeSurfer (http://surfer.nmr.mgh.harvard.edu); CT and SA were computed at each vertex. Details of image acquisition and processing are reported in the Supplementary Method.

### Statistical analyses

Mixed models regression analyses were used to characterize CT maturation over time at each vertex using MATLAB R2017a (MathWorks) (using code published at: https://github.com/danizoeller/myMixedModelsTrajectories) and GNU Parallel [29]. This method has been applied in previous studies by our group with a similar longitudinal design [23, 27, 30–32], as this approach is suitable for longitudinal datasets such as ours, with variability in the amount of scans per individual and in time between scans. Population parameters (group, age, and their interaction) were modeled as fixed effects, and subjects were modeled as random effects. The following equation was used to model CT as a function of age and group at each vertex:

$${\mathrm{Cortical}}\;{\mathrm{thickness}}\;{\mathrm{at}}\;{\mathrm{a}}\;{\mathrm{given}}\;{\mathrm{vertex}} = \beta _0 + \beta _{{\mathrm{g1}}} \ast {\mathrm{g}}_{\mathrm{i}} + \beta _{{\mathrm{a1}}} \ast {\mathrm{a}}_{{\mathrm{ij}}} + \\ \beta _{{\mathrm{ag1}}} \ast {\mathrm{g}}_{\mathrm{i}} \ast {\mathrm{a}}_{{\mathrm{ij}}} + \beta _{{\mathrm{a2}}} \ast {\mathrm{a}}^{\mathrm{2}}_{{\mathrm{ij}}} + \beta _{{\mathrm{ag2}}} \ast {\mathrm{g}}_{\mathrm{i}} \ast {\mathrm{a}}^2_{{\mathrm{ij}}} + {\mathrm{u}}_{{\mathrm{i0}}} + {\mathrm{u}}_{{\mathrm{i}}1} \ast {\mathrm{a}}_{{\mathrm{ij}}} + \varepsilon _{{\mathrm{ij}}},$$

where i = subject index; j = scan index; *β*_xn_ = fixed effects; g = grouping variable, a = age; u = normally distributed random effect; and *ε* = normally distributed error term.

Sex and scanner type were included as covariates. Intracranial volume (ICV) showed an association with SA but not with CT (Fig. S4). Therefore, ICV was added as a covariate only for SA analyses. Constant (no age-dependency, *β*_a1_ = *β*_ag1_ = *β*_a2_ = *β*_ag2_ = 0), linear (β_a2_ = β_ag2_ = 0) and quadratic (full equation above) random-slope models were fitted to the CT data at each vertex for the 22q11DS vs controls comparison; constant and linear models were fitted for the comparison of subgroups within 22q11DS to prevent overfitting in reduced samples. The best model order for a given vertex was determined using the Bayesian Information Criterion (BIC).

Then, the significance of group (intercept) and interaction (shape) effects were assessed using a log-likelihood ratio test comparing the full model described above with reduced models lacking group or age by diagnosis interaction terms, yielding respectively a model without group differences or with similar curve shapes in both groups. For all analyses, cluster-wise correction for multiple comparisons was applied at the significance threshold of *p* < 0.05 using Monte-Carlo permutation [33]. Result figures contain CT trajectories of the vertex with the largest effect within significant clusters.

Finally, to capture the timing of CT differences between groups (22q11DS vs controls; LA-PS vs N-PS), we computed snapshots of (1) the difference in CT between the estimated trajectories of each group, and (2) the difference in the annual rate of cortical thinning of each group at different ages. Corresponding videos showing dynamic CT and thinning differences between groups throughout development are available in the Supplementary Materials.

Additional analyses of SA following similar procedures are reported in the Supplementary Materials.

## Results

### Increased CT and accelerated thinning in 22q11DS

**Fig. 2:**
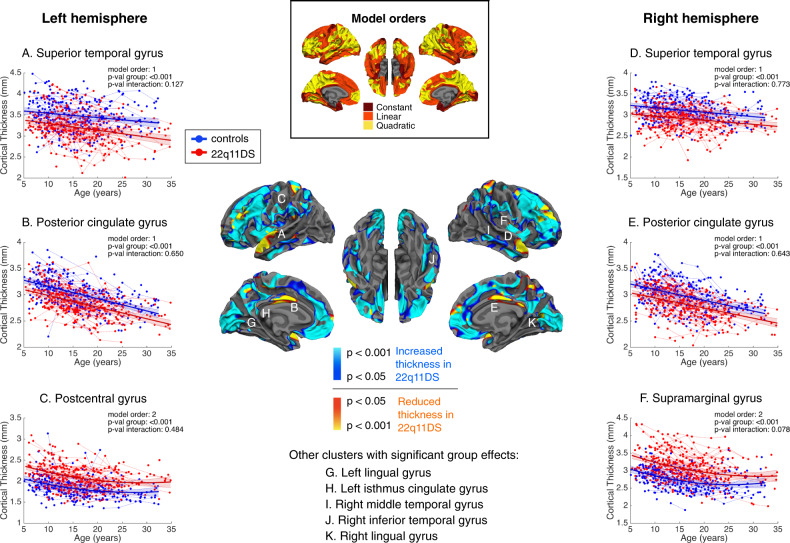
Significant intercept differences in cortical thickness between 22q11DS and controls after cluster-wise correction for multiple comparisons. In the central map, cold colors reflect increased cortical thickness, and warm colors indicate reduced cortical thickness in 22q11DS compared to controls. Individuals with 22q11DS show widespread increases in cortical thickness, with focal reductions in the superior temporal gyrus (STG) and in the posterior cingulate cortex (PCC). Representative thinning trajectories are displayed for the bilateral STG and PCC, the left postcentral gyrus, and the right supramarginal gyrus (legends (**A**)–(**F**)). For graphical representations of trajectories from other significant clusters (legends (**G**)–(**K**)), see Supplementary Fig. S5. The middle upper map depicts model orders fitted at each vertex, with dark red indicating constant models, orange corresponding to linear models, and yellow indicating quadratic models.

Vertex-wise mixed models regression of CT development in individuals with 22q11DS and controls yielded mostly linear (predominantly in frontal and temporal areas) or quadratic (prefrontal and parietal regions) model fits, with some constant model fits (occipital, precentral, and medial temporal regions). In both groups, CT trajectories were generally decreasing throughout childhood and adolescence, and either continued decreasing or leveled out at a minimum during adulthood (Fig. 2).

Group differences were widespread and involved higher CT in individuals with 22q11DS through most cortical regions, with cluster peaks located in the bilateral lingual gyri, left postcentral, and isthmus cingulate gyri, and right supramarginal, middle and inferior temporal gyri. Notable exceptions were observed in the bilateral posterior cingulate cortex (PCC) and STG, where CT was significantly lower in participants with 22q11DS compared to controls.

**Fig. 3:**
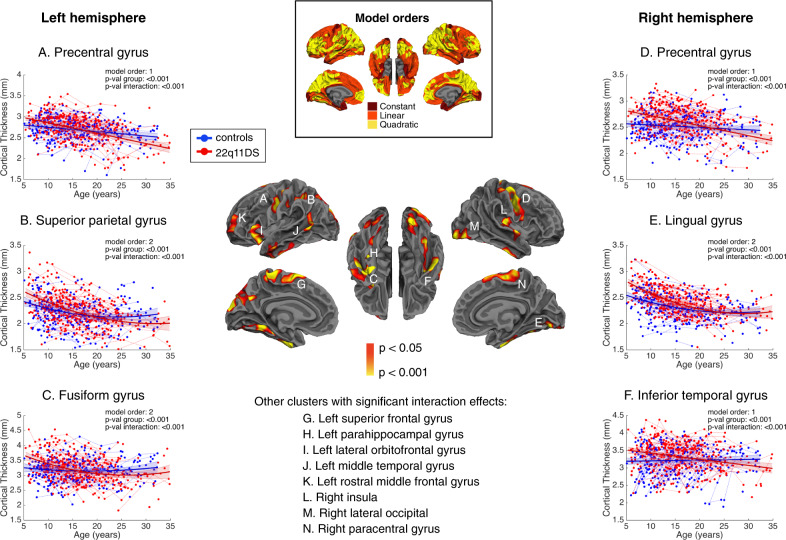
Significant shape differences in cortical thickness between 22q11DS and controls after cluster-wise correction for multiple comparisons. In the central map, warm colors reflect the degree of significance of shape differences found at each vertex. Individuals with 22q11DS show increased cortical thickness during childhood followed by accelerated thinning during adolescence, affecting most prominently frontotemporal regions. Representative thinning trajectories are displayed for the bilateral precentral gyrus, left superior parietal gyrus, left fusiform gyrus, right lingual gyrus, and right inferior temporal gyrus (legends (**A**)–(**F**)). For graphical representations of trajectories from other significant clusters (legends (**G**)–(**N**)), see Supplementary Fig. S6. The upper central map depicts model orders fitted at each vertex, with dark red indicating constant models, orange corresponding to linear models, and yellow indicating quadratic models.

Moreover, significant group by age interaction effects were evident in several brain regions, reflecting aberrant CT maturation in 22q11DS (Fig. 3). In these regions, participants with 22q11DS displayed abnormally higher CT values during childhood, followed by accelerated rates of cortical thinning, such that trajectories either converged towards normative development in adulthood or continued decreasing. Cluster peaks of altered developmental trajectories were predominantly found in frontotemporal regions including the left rostral middle, superior frontal, lateral orbitofrontal gyri, right paracentral, and bilateral precentral gyri as well left middle temporal, fusiform, and parahippocampal regions and the right inferior temporal gyrus. Additional clusters of altered CT trajectories were present in the left superior parietal and the right insula, lateral occipital, and lingual regions. Supplementary Table S3 and Figs. S5 and S6 contain detailed information of each of the clusters showing significant group or interaction effects.

**Fig. 4:**
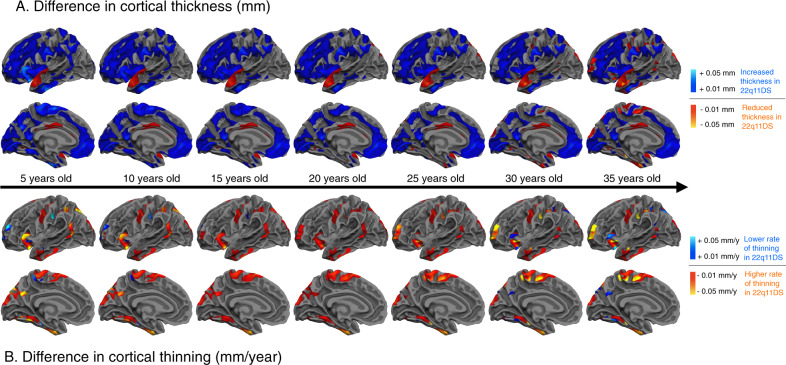
Time course of differences in cortical thickness and annual rate of cortical thinning between 22q11DS and controls in the left hemisphere. Cold colors reflect increased cortical thickness (**A**) or lower thinning rates (**B**) in 22q11DS compared to controls; warm colors indicate reduced cortical thickness (**A**) or higher thinning rates (**B**) in 22q11DS compared to controls. CT is increased throughout the cortex in 22q11DS compared to controls. CT differences tend to lessen during adolescence, although most differences remain evident throughout the brain in adulthood. Focal reductions in the posterior cingulate and superior temporal gyrus also remain present throughout development. Individuals with 22q11DS further show accelerated rates of thinning, most markedly in frontotemporal regions. Of note, thinning rates become more pronounced in certain regions when entering adulthood (e.g., left rostral middle frontal gyrus, superior frontal gyrus, paracentral gyrus, insula, supramarginal gyrus, superior parietal gyrus, parahippocampal gyrus, inferior temporal gyrus), reflecting a continued thinning process in individuals with 22q11DS. Videos displaying the evolution of cortical thickness and thinning rate differences between 22q11DS and controls overtime are available in the Supplementary Materials (Supplementary Video 1 for cortical thickness differences; Supplementary Video 2 for thinning rate differences).

Snapshots of differences in CT between both groups throughout the entire age range show left hemisphere regions in 22q11DS with a thicker cortex compared to controls (Fig. 4A). CT differences tended to lessen during adolescence, although most differences remained evident throughout the brain in adulthood. Differences in annualized thinning rates confirmed that individuals with 22q11DS had faster rates of cortical thinning compared to controls throughout childhood and adolescence (Fig. 4B). Interestingly, thinning rates became particularly exacerbated in certain regions when entering adulthood (e.g., left frontal and parietal regions, bilateral insula) reflecting a continued thinning process in individuals with 22q11DS that contrasted with healthy adults, where CT tended to stabilize. For corresponding brain maps of the right hemisphere, see Fig. S7; Supplementary Videos 1 and 2 show dynamic changes over time of CT and thinning rate differences between 22q11DS and controls.

### Exacerbated thinning in the STG in individuals with psychotic symptoms

Vertex-wise mixed models regression analyses comparing CT trajectories between LA-PS and N-PS participants within 22q11DS yielded mostly linear model fits, with some constant models in inferior and precentral areas.

**Fig. 5:**
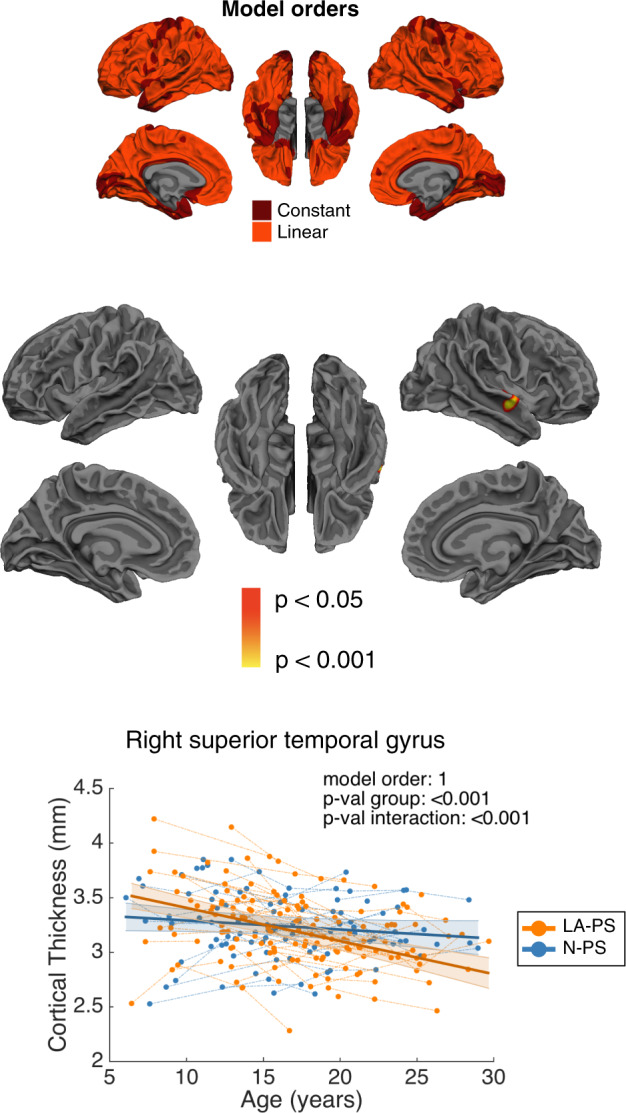
Brain maps showing regions with significant effects when comparing N-PS and LA-PS groups within 22q11DS. A significant interaction effect was found in the right superior temporal gyrus (STG), where individuals with positive psychotic symptoms showed steeper cortical thinning compared to individuals without psychotic symptoms. The upper map represents model orders fitted at each vertex, with dark red indicating constant models and orange corresponding to linear models.

No significant group differences were found between LA-PS and N-PS groups. Significant group by age interaction effects were observed in a cluster encompassing the right STG and extending to Heschl’s gyrus, where LA-PS individuals displayed accelerated cortical thinning compared to N-PS (Fig. 5).

Differences in CT and in annualized cortical thinning rates between LA-PS and N-PS individuals showed mostly reduced thickness and steeper thinning rates in LA-PS participants (Figs. S8 and S9). Supplementary Videos 3 and 4 show dynamic changes over time of CT and thinning rate differences between LA-PS and N-PS groups.

To further delineate CT maturation related to the development of psychosis, we conducted an exploratory analysis comparing three subgroups within 22q11DS (N-PS, LA-PS without psychosis, and LA-PS with psychosis). Interestingly, significant group-by-age interaction effects were again observed in the right STG, where LA-PS individuals with psychosis showed the steepest thinning trajectories compared to N-PS and LA-PS without psychosis (Fig. S10). An exacerbated thinning trajectory was further found in the left lateral occipital gyrus, and CT was significantly reduced in the right superior frontal gyrus for the LA-PS with psychosis group. Supplementary Tables S4 and S5 provide detailed results for the two and three group analyses within 22q11DS.

### SA trajectories

Vertex-wise mixed models regression of SA development comparing (1) individuals with 22q11Ds and controls and (2) LA-PS and N-PS individuals within 22q11DS are reported in the Supplementary Results and Tables S6 and S7. Trajectories of SA were mostly constant, reflecting an absence of SA changes during the included age range. SA was reduced in most cortical areas in 22q11DS, except for precentral areas showing focal increases (Fig. S11). One significant group by age interaction effect was evident in the left superior parietal region, where controls showed a gradual SA decline, while SA levels remained constant in individuals with 22q11DS (Fig. S12).

SA trajectories comparing LA-PS and N-PS participants within 22q11DS yielded group differences in several regions, with increases in temporal and parietal areas, as well as decreased SA in the superior parietal area (Fig. S13). No significant group-by-age interaction effects were observed.

## Discussion

This study represents the largest longitudinal investigation of CT and SA maturation in 22q11DS to date, providing a delineation of neuroanatomical trajectories from childhood to adulthood with exquisite spatial and temporal resolution. Overall, the current study demonstrated a widespread pattern of continuously higher CT in individuals with 22q11DS, with the notable exception of focal reductions in PCC and STG. Moreover, several predominantly frontotemporal areas displayed deviant trajectories characterized by higher CT during childhood in 22q11DS, followed by accelerated rates of cortical thinning during adolescence resulting in CT values similar to, or lower than those of adult controls. Analyses comparing CT in N-PS *vs* LA-PS groups within 22q11DS further revealed steeper thinning rates in the right STG of participants experiencing positive psychotic symptoms.

In line with recent surface-based studies in 22q11DS [14–17, 19], our longitudinal analysis confirmed the presence of a widespread thicker cortex in 22q11DS. On a neurobiological level, abnormally increased CT may result from disruptions in early, prenatal developmental events involving cell proliferation and/or migration. Given that CT is thought to reflect cell density within cortical columns [34], increased CT may indicate an over-proliferation of intermediate progenitor cells during corticogenesis. Furthermore, post-mortem examinations have reported a high incidence of periventricular heterotopias suggestive of abnormal neuronal migration [35], and abnormal migration of interneurons has been confirmed in a mouse model of 22q11DS [36].

22q11DS was further characterized by focal reductions bilaterally in the PCC and anterior STG, a finding that was observed in several previous studies in 22q11DS [14, 15, 17]. These persistent reductions suggest different early neurodevelopmental anomalies in these areas, such as a deficit in intermediate progenitor cell proliferation. Future studies in mouse models of 22q11DS are needed to understand the mechanisms underlying localized CT reductions, as compared to thicker CT in most other cortical areas.

Regarding maturational trajectories, our finding of widespread cortical thinning from childhood to adulthood is in line with recent reports on typical neurodevelopment describing continuous thinning throughout early childhood and adolescence (for a review, see [37]). Cortical thinning during adolescence has been proposed to reflect synaptic pruning, i.e., the elimination of unused synapses and the refinement of neuronal network connections. More recently, studies have demonstrated that other mechanisms including myelination (causing the apparent gray-white matter boundary to shift outwards) and morphological changes (causing the neuropil to “stretch”), or a combination of these mechanisms, may possibly also underlie cortical thinning captured by structural MRI [38, 39].

These findings are especially interesting given the presence of accelerated rates of cortical thinning during adolescence and early adulthood in 22q11DS compared to controls found in our study. Supporting evidence for aberrant synaptic plasticity in 22q11DS comes from studies on LgDel mouse models of the syndrome showing impaired synaptic spine stabilization [40] and reduced synaptic plasticity in parvalbumin expressing interneurons, which have been involved in the neurobiology of schizophrenia [41]. CT alterations in 22q11DS have also been associated with P2RX6, a 22q11 gene involved in synaptic signaling [42]. In parallel, recent longitudinal studies have provided evidence for potentially excessive myelination [43] and aberrant development of structural morphology in 22q11DS [24]. Therefore, it is possible that the genetic vulnerability for psychosis conveyed by the deletion involves anomalies in multiple tightly regulated developmental mechanisms. For a detailed discussion on potential genetic mechanisms involved in abnormal brain development in 22q11DS, see [44].

Accelerated rates of cortical thinning in 22q11DS were mostly found in frontotemporal regions, in line with previous findings in 22q11DS [19]. Interestingly, thinning in 22q11DS became even more deviant in adulthood in several frontal, medial temporal, and parietal structures, as well as in the insula. In these structures, individuals with 22q11DS continued to exhibit thinning, whereas CT tended to stabilize in controls during adulthood (25–35 years), suggesting that syndromic individuals may show early aging-related thinning [45]. Given that aging has been less investigated in 22q11DS, it will be important to determine the cognitive and psychiatric outcomes associated with continued thinning during adulthood.

Of note, while CT differences lessened somewhat as participants reached adulthood, several brain regions remained markedly increased. This contrasts with typical neurodevelopment, where particularly prefrontal regions are known to undergo pronounced forms of pruning [46]. While further human and animal studies are needed to identify the neurobiological underpinnings of this finding, we can speculate that these regions may not undergo efficient synaptic elimination in the syndrome.

Within 22q11DS, participants experiencing positive psychotic symptoms showed steeper cortical thinning in the right STG, but not in other regions. While the specific involvement of the STG contrasts with previous reports indicating developmental alterations in frontal [21–23], cingulate [23] or parietal areas [23, 24] in patients with 22q11DS experiencing psychotic symptoms, it is consistent with a growing body of evidence in 22q11DS suggesting a critical involvement of temporal areas in the emergence of psychosis [21, 44, 47]. Reductions in the right STG have also been reported in meta-analyses of schizophrenia [6] and ultra-high-risk individuals who develop psychosis [48] and have been associated with positive psychotic symptoms in a multisite study on idiopathic schizophrenia [49], pointing to a critical involvement of this brain structure in the pathophysiology of the disease in both the general population and individuals with a genetic vulnerability. Our exploratory three-group analysis further suggested that individuals with LA-PS who develop overt psychosis show the steepest thinning rates in this region, compared to LA-PS without psychosis and N-PS groups. These findings will however need confirmation in larger longitudinal samples.

Importantly, many of the developmental findings in 22q11DS found in this study corroborate anomalies reported in idiopathic schizophrenia, including thinner PCC and anterior STG [25, 50], and exacerbated thinning in frontotemporal areas [9, 51]. However, only thinning in the STG was associated with positive symptoms in 22q11DS in this study. We can speculate that the genetic burden caused by 22q11DS leads to generally disrupted developmental mechanisms, starting with prenatal proliferation/migration, followed by postnatal synaptic pruning, myelination, and/or morphology alterations. The STG may be a particularly vulnerable area that, when subjected to maturational disruptions, triggers the emergence of psychotic symptoms. Alternatively, neurodevelopmental disruptions in other areas may be associated with specific symptom profiles. Evidence for symptom-specific brain anomalies comes from a recent study on a partially overlapping sample, where we showed that in individuals with 22q11DS experiencing auditory hallucinations, functional hyperconnectivity between the anterior STG and the medial geniculate nucleus (MGN) of the thalamus was associated with MGN atrophy [52]. A promising future direction would be to investigate whether neuroanatomical alterations map onto specific symptom profiles.

Taken together, the current findings suggest that while global CT increases are likely a hallmark of 22q11DS caused by early developmental anomalies, localized accelerated thinning in the STG during adolescence may represent a valuable early biomarker related to the emergence of psychotic symptoms.

Widely decreased SA in 22q11DS compared to controls found in our analyses is in line with previous studies reporting reduced SA in 22q11DS using parcellation [15] and vertex-wise cross-sectional approaches [14, 16, 17]. For a detailed discussion of SA findings, see Supplementary Discussion.

The main limitation of this study concerns the use of scanners with different field strengths (*N* = 158 1.5 T scans, *N* = 478 3 T scans), a frequent limitation in longitudinal cohorts where data collection spans through a large time period. Nevertheless, we have previously demonstrated high cross-scanner reliability in structural measures [32] and scanner was added as a covariate to control for potential confounding effects. Next, while this study comprised the largest longitudinal analysis reported thus far, the sample available for the analysis of subgroups within 22q11DS was limited. Future longitudinal studies including larger samples of participants with psychotic symptoms, particularly individuals developing overt psychosis, are warranted to confirm our findings. Finally, data used in this study did not provide means to determine the mechanisms (i.e., aberrant corticogenesis, pruning, myelination, morphology) responsible for aberrant CT in 22q11DS and in the emergence of psychosis. The investigation of brain maturation using animal models and human multimodal studies including, for instance, the T1/T2 ratio [37, 53] allowing to measure intracortical myelination, will be important to understand the mechanisms underlying CT changes.

Overall, the findings of the current study confirm the existence of neurodevelopmental alterations in a population at high genetic risk for psychosis and underline the importance of taking a developmental perspective when examining neuropathological mechanisms of this illness. For the first time, this study provided fine-grained delineations of developmental trajectories, revealing critical information regarding the timing and potential mechanisms underlying neurodevelopmental disruptions in 22q11DS. While future studies are warranted, current evidence suggests a combination of early prenatal alterations leading to the thicker cortex in 22q11DS, combined with later maturational anomalies involving excessive thinning during adolescence, a mechanism that may specifically affect the right STG in the onset of positive psychotic symptoms.

## Supplementary information

Supplementary Video 1

Supplementary Video 2

Supplementary Video 3

Supplementary Video 4

Supplementary Material
