# Supplementary material for: Altered cortical thickness development in 22q11.2 deletion syndrome and association with psychotic symptoms
Source: Mol Psychiatry. 2021 Jul 12;26(12):7671–8. doi: 10.1038/s41380-021-01209-8 (PMC8873018; doi:10.1038/s41380-021-01209-8)
Supplement: Supplementary file 5 — Supplementary Material [file 41380_2021_1209_MOESM5_ESM.docx]

**Supplementary Materials for: Altered cortical thickness development in 22q11.2 deletion syndrome and association with psychotic symptoms**

J. Bagautdinova, D. Zöller, M. Schaer, M.C. Padula, V. Mancini, M. Schneider, S. Eliez

Table of Contents

[Supplementary Method 3](#_Toc74678016)

[Participants 3](#_Toc74678017)

[Neurocognitive measures 4](#_Toc74678018)

[Psychiatric assessment 4](#_Toc74678019)

[Image acquisition details 5](#_Toc74678020)

[Image exclusion criteria 5](#_Toc74678021)

[Image processing details 5](#_Toc74678022)

[Supplementary Results 7](#_Toc74678023)

[Constant trajectories of decreased surface area in 22q11DS 7](#_Toc74678024)

[Increased temporal and decreased parietal surface area in individuals with psychotic symptoms within 22q11DS 7](#_Toc74678025)

[Supplementary Discussion 8](#_Toc74678026)

[Supplementary Tables 10](#_Toc74678027)

[Table S1. 10](#_Toc74678028)

[Table S2. 11](#_Toc74678029)

[Table S3. 12](#_Toc74678030)

[Table S4. 13](#_Toc74678031)

[Table S5. 13](#_Toc74678032)

[Table S6. 14](#_Toc74678033)

[Table S7. 14](#_Toc74678034)

[Supplementary Figures 15](#_Toc74678035)

[Figure S1. 15](#_Toc74678036)

[Figure S2. 16](#_Toc74678037)

[Figure S3. 17](#_Toc74678038)

[Figure S4. 18](#_Toc74678039)

[Figure S5. 19](#_Toc74678040)

[Figure S6. 20](#_Toc74678041)

[Figure S7. 21](#_Toc74678042)

[Figure S8. 22](#_Toc74678043)

[Figure S9. 23](#_Toc74678044)

[Figure S10. 24](#_Toc74678045)

[Figure S11. 25](#_Toc74678046)

[Figure S12. 26](#_Toc74678047)

[Figure S13. 27](#_Toc74678048)

[References 28](#_Toc74678049)

## Supplementary Method

### Participants

Participants were recruited through parent associations and word of mouth for the Geneva longitudinal 22q11DS study since 2001 (1,2).

Descriptive statistics of sample demographics, age, gender and IQ differences between groups were computed using R (http://www.R-project.org/).

For the modeling of cortical thickness (CT) development in 22q11DS and controls, the sample consisted of 324 participants (N=148 22q11DS (73 male, 75 female); N = 176 controls (90 male, 86 female)) aged 5-35 years who contributed between 1-6 scans, resulting in a total of 636 scans (N=334 22q11DS (153 male, 181 female); N = 302 controls (143 male, 159 female), see Figure S1A). For individuals with multiple time points, mean time interval between scans was M=3. 571 years (SD=1.113), mean total follow-up time between the first and last scan of the same individual was M=6.713 (SD=3.728). There was no significant difference in gender (p=0.745) or age (p=0.319) between patients with 22q11DS and controls. There was a significant difference in full-scale IQ (p<0.001), which can be expected as individuals with 22q11DS have a full scale IQ of around 70-75 on average (3). Table S1 contains a summary of the above description of patients with 22q11DS and controls.

For the modeling of CT trajectories in subgroups of patients presenting lifetime attenuated positive psychotic symptoms (LA-PS) *vs* no positive psychotic symptoms (N-PS), the sample consisted of 108 patients with 22q11DS (N=47 N-PS (24 male, 23 female); N=61 LA-PS (32 male, 29 female)) aged 6-28 years who contributed between 1-5 scans, resulting in a total of 244 scans (N=98 N-PS (48 male, 50 female); N=146 LA-PS (68 male, 78 female), see Figure S1B). For individuals with multiple time points, mean time interval between scans was M=3.89 years (SD=1.314) and mean total follow-up time between the first and last scan of the same individual was M=7.15 (SD=3.305). There was no significant difference in gender (p=0.886), age (p=0.231) or full-scale IQ (p=0.22) between N-PS and LA-PS patients. Table S1 contains a summary of the above description of subgroups of patients with 22q11DS.

### Neurocognitive measures

Full-scale IQ was evaluated using age-adapted versions of the Wechsler intelligence scale (i.e. the Wechsler Intelligence Scale for Children, version III or IV, or the Wechsler Adult Intelligence Scale, version III or IV) (4–7).

### Psychiatric assessment

The presence of psychiatric disorders was assessed using the Diagnostic Interview for Children and Adolescents Revised (DICA-R) (8), the psychosis supplement from the Kiddie-Schedule for Affective Disorders and Schizophrenia Present and Lifetime version (K-SADS-PL) (9), and the Structured Clinical Interview for DSM-IV Axis I Disorders (SCID-I) (10) for adult patients (starting from 18 years).

### Image acquisition details

T1-weighted MRI anatomical brain scans were acquired using three different scanners: a 1.5T Philips Intera scanner (158 scans), a 3T Siemens Trio scanner (300 scans) and a 3T Siemens Prisma scanner (178 scans). The 1.5T Philips Intera scanner was used from 2002-2007, the 3T Siemens Trio scanner from 2007-2014 and the 3T Siemens Prisma scanner from 2014 up to the present date. The combination of multiple different scanners is inherent to large longitudinal cohorts such as ours where data collection has been ongoing since 19 years and is also increasingly common in large-scale multi-site studies (11–13). Similar to these studies, we have addressed this issue by including scanners as a covariate in the analyses. Importantly, previous studies have shown that cortical thickness measurements are reliable, independently of scanner manufacturer or field strength (14). Moreover, we previously quantified the difference in cortical thickness estimation at each cortical point for 20 healthy participants of this sample that underwent MRI acquisitions with the two scanners on the same day, showing very high cross-scanner consistency in 90% of the cortical surface (see Method & Supplementary Figure 1 in (15)).

For the 1.5T Philips Intera scanner, the sequence was composed of 124 coronal slices with a voxel size of 0.94x0.94x1.5mm (TR=35 ms, TE=6 ms, flip angle=45°, matrix size=256x192, field of view=24 cm^2^). On the 3T Siemens Trio and Prisma scanners, the sequence comprised 192 coronal slices with voxel size of 0.86x0.86x1.1 mm (TR=2500 ms, TE=3 ms, flip angle=8°, acquisition matrix=224×256, field of view=22 cm^2^).

### Image exclusion criteria

All scans of good quality (e.g., no excessive motion or artifacts) and without visible anomalies were included. Two participants with polymicrogyria and one participant with signs of perinatal stroke were excluded.

### Image processing details

Anatomical segmentation of T1-weighted images was performed using FreeSurfer (http://surfer.nmr.mgh.harvard.edu), with the goal to reconstruct accurate three-dimensional representations of the inner and outer surfaces of the cortical mantle with sub-millimeter accuracy (16,17). Automated image preprocessing included resampling into cubic voxels, intensity normalization and skull stripping. Reconstruction of the white (gray-white boundary) and pial (gray-CSF interface) cortical surfaces used deformation algorithms based on the local intensity value, geometrical and topological constraints. The obtained cortical mesh models were subsequently used for measurements of cortical thickness (CT), defined as the distance between the white (grey-white boundary) and pial (grey-CSF interface) surfaces, as well as cortical surface area (SA) measured at the grey/white matter boundary. At the end of the reconstruction process, CT values with a submillimeter accuracy were available at 163’842 vertices by hemisphere. Segmentation accuracy was manually reviewed and corrected where necessary.

Inter-subject comparison of CT and SA maps was then achieved through spherical registration of the surfaces to the *fsaverage* subject included in FreeSurfer, allowing reliable point-to-point comparisons of CT and SA across all scans (18,19). Surface-based smoothing of CT and SA data was done in the template’s space using a FWHM of 10 mm.

To further verify the quality of CT outputs, we obtained the standardized CT distribution at each vertex, flagging scans with a CT value of more than 3 standard deviations from the norm for a particular vertex. Scans were considered outliers if they had more than 10% of vertices meeting the above-mentioned criterion. As scans selected for the study contained a maximum of 8.9% outlier vertices, all scans were considered of sufficient quality and were included in the analyses.

## Supplementary Results

### Constant trajectories of decreased surface area in 22q11DS

Vertex-wise mixed models regression of SA development in individuals with 22q11DS and controls yielded mostly constant model fits, reflecting an absence of SA changes during the included developmental period.

Group differences were widespread and involved reduced SA in individuals with 22q11DS through most cortical regions, with cluster peaks located in the left precuneus and right rostral middle frontal gyrus. Focal increased SA was observed bilaterally in the precentral gyri (Figure S11).

Moreover, significant group by age interaction effects were evident in the left superior parietal region (Figure S11). In this region, controls showed gradual decreases in SA during development, while SA levels remained constant in individuals with 22q11DS. Supplementary Table S6 contains clusters with significant group and interaction effects. Given that most cortical areas followed constant trajectories with no change in SA, figures or videos of SA changes throughout development were not relevant for this metric.

### Increased temporal and decreased parietal surface area in individuals with psychotic symptoms within 22q11DS

Vertex-wise mixed models regression analyses comparing SA trajectories between LA-PS and N-PS participants within 22q11DS yielded a majority of constant model fits, indicating that SA measures mostly did not show developmental changes during the time period considered.

Significant group differences were observed in several regions. Surface area was increased in the LA-PS group in the bilateral middle temporal gyri and in the right inferior parietal region. By contrast, SA was decreased in the right superior parietal gyrus in LA-PS compared to N-PS individuals (Supplementary Table S7, Figure S13).

No significant group by age interaction effects were observed.

## Supplementary Discussion

While CT has received most interest in the context of brain maturation and alterations leading to psychosis, surface area (SA) is another potentially important, yet understudied component of brain structure. The additional analyses of SA trajectories performed in this study revealed widespread decreased SA in 22q11DS compared to controls, in line with previous studies reporting reduced SA in 22q11DS using parcellation (20) and vertex-wise cross-sectional approaches (12,21,22). Focally increased SA was found bilaterally in precentral regions in our study, a finding that has also been reported in a prior large-scale, vertex-wise analysis in 22q11DS (12).

SA analyses further indicated largely constant SA levels in 22q11DS and controls, indicating an absence of SA changes during the included age range. Only the superior parietal area showed a deviant developmental pattern, with controls showing gradual decreases in SA during development, while SA levels remained constant in individuals with 22q11DS. To date, few vertex-wise cross-sectional studies have characterized age-related SA changes in 22q11DS, and generally reported steeper SA decline rates over time in syndromic individuals (21) or linear to constant trajectories in both 22q11DS and controls (12). Longitudinal studies on typical developing controls including various age ranges and using whole-brain averages or parcellation approaches reported mixed results including both linear decreases (7-29 years, (23)) and non-linear increases in SA (11-20 years, (24); 7-23years, (25); 3-30 years, (26)). By contrast, the only vertex-wise study performed to date on typical developing individuals indicated that most brain regions showed no change in SA metrics between 5-22 years (27). Results of the current longitudinal study support the presence of mostly constant SA levels during the age range considered (5-35 years), both in 22q11DS and in typical developing controls.

On a biological level, CT and SA are governed by distinct neurodevelopmental processes. More specifically, the radial unit hypothesis proposes that during development, CT is determined by the number of cells within a cortical column, while SA expansion is influenced by the number of columns produced during corticogenesis (28,29). Given that 22q11DS is associated with widespread SA reductions visible as early as five years of age, it is plausible that the deletion involves early disruptions in the production of radial unit progenitors, which then persist throughout the course of postnatal development. Support for this hypothesis comes from a recent genetic association study, which found that SA reductions in 22q11DS were associated with the expression of 22q11 genes involved in cell cycle regulation (DGCR8, AIFM3) (30).

Analyses comparing SA in N-PS *vs* LA-PS groups within 22q11DS revealed a somewhat unexpected pattern of increased SA in temporal areas, combined with decreased SA in the superior parietal gyrus in individuals with positive psychotic symptoms. This is in contrast with prior evidence in 22q11DS mostly reporting no SA differences in patients with psychotic symptoms (12,20–22,31), as well as findings in idiopathic schizophrenia reporting an overall uniform pattern of SA reductions (32–34). However, trend level associations have been reported between SA and psychotic symptoms in 22q11DS (20) and one longitudinal study found a similar pattern of mixed reduced and increased SA in UHR individuals with 22q11DS (35). Moreover, a longitudinal study found increased SA in familial high-risk individuals transitioning to psychosis (36). It is therefore possible that SA alterations may generally remain undetected in smaller cross-sectional samples, and/or may present a more heterogeneous pattern in stages preceding psychosis.

While further investigations are warranted, the large-scale longitudinal, vertex-wise approach of the current study suggests that 1) SA levels remain stable in most regions between 5-35 years in both 22q11DS and typical developing controls; 2) SA alterations associated with 22q11DS occur early (before age five), and 3) some SA alterations are associated with the presence of positive psychotic symptoms. The mechanisms underlying SA alterations remain to be uncovered, but evidence in both the 22q11DS group and the subgroup with psychotic symptoms suggests that the observed SA anomalies result from early neurodevelopmental events. Future studies should investigate early developmental stages and should use translational approaches to further delineate SA maturation during prenatal and perinatal development in rodent models of 22q11DS.

## Supplementary Tables

Table S1. Demographic information for 22q11DS and controls and for subgroups of individuals with 22q11DS with (LA-PS) or without (N-PS) positive psychotic symptoms.

|  | | | | | | |
| --- | --- | --- | --- | --- | --- | --- |
|  | 22q11DS | Healthy controls | p-value | 22q11DS  N-PS | 22q11DS  LA-PS | p-value |
|  | | | | | | |
| N subjects (% female) | 148 (50.68%) | 176 (58.11%) |  | 47 (48.94%) | 61 (47.54%) |  |
| N with 1 visit | 58 | 99 |  | 19 | 15 |  |
| N with 2 visits | 36 | 44 |  | 12 | 19 |  |
| N with 3 visits | 25 | 20 |  | 10 | 16 |  |
| N with 4 visits | 17 | 10 |  | 5 | 10 |  |
| N with 5 visits | 11 | 2 |  | 1 | 1 |  |
| N with 6 visits | 1 | 1 |  | 0 | 0 |  |
| N scans (total) | 334 | 302 |  | 98 | 146 |  |
| N per scanner (1.5T/3T Trio/3T Prisma) | 75/153/106 | 83/147/72 | 0.066 | 15/45/38 | 19/72/55 | 0.827 |
| Mean FSIQ | 71.24 ± 12.42 | 110.75 ± 13.32 | < 0.001 | 72.83 ± 12.12 | 71.90 ± 14.21 | 0.22 |
| Age range (years) | 5.40-34.82 | 5.11-32.35 |  | 6.06-28.43 | 6.43-25.45 |  |
| Mean age (years) | 16.47 ± 6.37 | 15.96 ± 6.56 | 0.319 | 13.54 ± 4.64 | 13.86 ± 4.29 | 0.231 |
| Mean age at first visit (years) | 13.69 ± 6.52 | 14.59 ± 6.93 | 0.230 | 13.54 ± 4.64 | 13.86 ± 4.29 | 0.711 |
| Mean time interval between visits (years) | 3.58 ± 1.20 | 3.55 ± 0.97 | 0.792 | 4.08 ± 1.46 | 3.77 ± 1.22 | 0.203 |
| Total follow-up time (years) | 7.41 ± 3.77 | 5.89 ± 3.52 | 0.008 | 7.44 ± 3.25 | 6.97 ± 3.36 | 0.556 |
| N medicated | 92 (62.16%) | - |  | 31 (65.96%) | 41 (67.21%) |  |
| Methylphenidate | 53 (35.81%) | - |  | 24 (51.06%) | 16 (26.23%) |  |
| Antidepressants | 39 (26.35%) | - |  | 10 (21.28%) | 25 (40.98%) |  |
| Antipsychotics | 26 (17.57%) | - |  | 2 (4.26%) | 18 (29.51%) |  |
| Anxiolytics | 16 (10.81%) | - |  | 1 (2.13%) | 9 (14.75%) |  |
| Antiepileptics | 11 (7.43%) | - |  | 2 (4.26%) | 6 (9.84%) |  |
| More than one type of medication | 36 (24.32%) | - |  | 7 (14.89%) | 21 (34.43%) |  |
| N with psychiatric diagnosis | 125 (84.46%) | - |  | 36 (76.60%) | 57 (93.44%) |  |
| ADHD | 70 (47.30%) | - |  | 22 (46.81%) | 34 (55.74%) |  |
| Anxiety disorder | 96 (64.86%) | - |  | 24 (51.06%) | 52 (85.25%) |  |
| Mood disorder | 63 (42.57%) | - |  | 16 (34.04%) | 32 (52.46%) |  |
| Psychotic disorder | 21 (14.19%) | - |  | 0 | 12 (19.67%) |  |
| OCD | 15 (10.14%) | - |  | 4 (8.51%) | 7 (11.48%) |  |
| More than one diagnosis | 88 (59.46%) | - |  | 26 (55.32%) | 48 (78.69%) |  |
|  | | | | | | |

Table S2. Mixed model results assessing the effects of age, diagnosis and scanner on mean cortical thickness (averaged across both hemispheres). Age and diagnosis have a significant effect on mean cortical thickness; scanner type did not have a significant effect.

|  | | | | | | |
| --- | --- | --- | --- | --- | --- | --- |
|  | **Sum Sq** | **Mean Sq** | **NumDF** | **DenDF** | **F value** | **Pr(> F)** |
|  | | | | | | |
| age | 2.893 | 2.893 | 1 | 515.777 | 740.251 | <0.001 |
| typescan | 0.016 | 0.008 | 2 | 509.383 | 2.110 | 0.122 |
| diagnosis | 0.191 | 0.191 | 1 | 309.772 | 48.817 | <0.001 |
|  | | | | | | |

Table S3. Maximum values of significant clusters after cluster-wise correction for multiple comparison of mixed models comparing cortical thickness in 22q11DS vs controls using the *mri_surfcluster* function from FreeSurfer. Gender and scanner were included as covariates in the analyses. Max = maximum -log10(p-value) found in the cluster. Size (mm^2^) = surface area of the cluster. Tal(X,Y,Z) = MNI coordinates of the maximum p-value. CWP = cluster-wise p-value.

| **Hemi-sphere** | **Model effect** | **Brain**  **Region** |  | **Cluster Number** | **Max** | **Size (mm^2^)** | **TalX** | **TalY** | **TalZ** | **CWP** |
| --- | --- | --- | --- | --- | --- | --- | --- | --- | --- | --- |
| Left | Group effects | Postcentral |  | 1 | -307.653 | 24126.94 | -54.7 | -10.6 | 29.4 | 0.0001 |
|  |  | Lingual |  | 2 | -307.653 | 6382.87 | -7.7 | -77.3 | 2.2 | 0.0001 |
|  |  | Isthmus cingulate |  | 3 | -11.695 | 2054.02 | -6.8 | -53.2 | 11.2 | 0.0001 |
|  |  | Superior temporal |  | 4 | 10.954 | 1446.48 | -50.7 | 3.4 | -17 | 0.0001 |
|  |  | Posterior cingulate |  | 5 | 9.266 | 486.58 | -4.2 | -1.1 | 33.2 | 0.0325 |
|  | Interaction effects | Fusiform |  | 1 | 5.511 | 1610.32 | -32.4 | -9.2 | -27.1 | 0.0001 |
|  |  | Precentral |  | 2 | 5.1 | 1812.82 | -43.1 | -9.8 | 36.3 | 0.0001 |
|  |  | Superior parietal |  | 3 | 4.757 | 2130.62 | -11.5 | -88.8 | 20.3 | 0.0001 |
|  |  | Superior frontal |  | 4 | 4.537 | 630.08 | -6.6 | 2.8 | 59.5 | 0.0051 |
|  |  | Parahippocampal |  | 5 | 4.364 | 708.23 | -34.7 | -29.8 | -13.4 | 0.0024 |
|  |  | Lateral orbitofrontal |  | 6 | 3.735 | 716.08 | -29.7 | 25.6 | -8.9 | 0.0021 |
|  |  | Superior parietal |  | 7 | 3.732 | 839.86 | -32.6 | -44 | 44.6 | 0.0005 |
|  |  | Middle temporal |  | 8 | 3.437 | 623.44 | -57.9 | -57.3 | 5.9 | 0.0055 |
|  |  | Rostral middle frontal |  | 9 | 2.863 | 772.79 | -22.3 | 49.8 | -1.3 | 0.0011 |
|  |  | Fusiform |  | 10 | 2.406 | 556.31 | -35.8 | -73.1 | -8.5 | 0.0129 |
| Right | Group effects | Supramarginal |  | 1 | -307.653 | 28740.39 | 52.9 | -24.2 | 36.5 | 0.0001 |
|  |  | Lingual |  | 2 | -307.653 | 3796.32 | 4.4 | -81.2 | 1.2 | 0.0001 |
|  |  | Superior temporal |  | 3 | 9.897 | 874.91 | 50 | -2.2 | -10.4 | 0.0001 |
|  |  | Middle temporal |  | 4 | -9.596 | 1517.17 | 50.2 | -2.9 | -26 | 0.0001 |
|  |  | Posterior cingulate |  | 5 | 7.621 | 525.08 | 3.9 | -6.1 | 33.3 | 0.0227 |
|  |  | Inferior temporal |  | 6 | -6.272 | 1076.95 | 43.1 | -57.4 | -5.1 | 0.0001 |
|  | Interaction effects | Precentral |  | 1 | 6.239 | 2048.93 | 44.8 | -6.4 | 31.7 | 0.0001 |
|  |  | Lingual |  | 2 | 5.206 | 896.19 | 18.6 | -76.8 | -5.8 | 0.0001 |
|  |  | Inferior temporal |  | 3 | 4.243 | 1152.39 | 44.4 | -12.4 | -30.9 | 0.0001 |
|  |  | Insula |  | 4 | 4.182 | 1217.77 | 33 | -20.4 | 12.4 | 0.0001 |
|  |  | Lateral occipital |  | 5 | 3.732 | 1039.62 | 27.1 | -94.1 | -4.1 | 0.0001 |
|  |  | Paracentral |  | 6 | 3.593 | 936.08 | 6.4 | -9.9 | 57.8 | 0.0001 |

Table S4. Maximum values of significant clusters after cluster-wise correction for multiple comparison of mixed models comparing cortical thickness in N-PS *vs* LA-PS individuals with 22q11DS using the *mri_surfcluster* function from FreeSurfer. Gender and scanner were included as covariates in the analyses. Max = maximum -log10(p-value) found in the cluster. Size (mm^2^) = surface area of the cluster. Tal(X,Y,Z) = MNI coordinates of the maximum p-value. CWP = cluster-wise p-value.

| **Hemi-sphere** | **Model effect** | **Brain**  **Region** | **Cluster Number** | **Max** | **Size (mm^2^)** | **TalX** | **TalY** | **TalZ** | **CWP** |
| --- | --- | --- | --- | --- | --- | --- | --- | --- | --- |
| Right | Interaction effects | Superior temporal | 1 | 4.288 | 552.82 | 56.7 | -9 | -5.6 | 0.0166 |

Table S5. Maximum values of significant clusters after cluster-wise correction for multiple comparison using the *mri_surfcluster* function from FreeSurfer for mixed models comparing cortical thickness in three subgroups within 22q11DS: N-PS individuals, LA-PS individuals without psychosis and LA-PS individuals with psychosis. Gender and scanner were included as covariates in the analyses. Max = maximum -log10(p-value) found in the cluster. Size (mm^2^) = surface area of the cluster. Tal(X,Y,Z) = MNI coordinates of the maximum p-value. CWP = cluster-wise p-value.

| **Hemi-sphere** | **Model effect** | **Brain**  **Region** | **Cluster Number** | **Max** | **Size (mm^2^)** | **TalX** | **TalY** | **TalZ** | **CWP** |
| --- | --- | --- | --- | --- | --- | --- | --- | --- | --- |
| Left | Interaction effects | Lateral occipital | 1 | 3.510 | 495.20 | -26.9 | -82.0 | -8.1 | 0.02970 |
| Right | Group effects | Superior frontal | 1 | 3.277 | 605.41 | 21.8 | 0.9 | 47.6 | 0.00840 |
|  | Interaction effects | Superior temporal | 1 | 4.510 | 678.06 | 48.1 | -16.2 | -2.3 | 0.00310 |

Table S6. Maximum values of significant clusters after cluster-wise correction for multiple comparison of mixed models comparing surface area 22q11DS vs controls using the *mri_surfcluster* function from FreeSurfer. Gender, scanner and intracranial volume were included as covariates in the analyses. Max = maximum -log10(p-value) found in the cluster. Size (mm^2^) = surface area of the cluster. Tal(X,Y,Z) = MNI coordinates of the maximum p-value. CWP = cluster-wise p-value.

| **Hemi-sphere** | **Model effect** | **Brain**  **Region** | **Cluster Number** | **Max** | **Size (mm^2^)** | **TalX** | **TalY** | **TalZ** | **CWP** |
| --- | --- | --- | --- | --- | --- | --- | --- | --- | --- |
| Left | Group effects | Precuneus | 1 | 307.653 | 56828.80 | -21.2 | -58.9 | 11.2 | 0.00010 |
|  |  | Precentral | 2 | -5.827 | 611.01 | -17.5 | -11.7 | 56.2 | 0.00680 |
|  | Interaction effects | Superior parietal | 1 | 2.532 | 459.47 | -25.8 | -69.3 | 22.9 | 0.04770 |
| Right | Group effects | Rostral middle frontal | 1 | 307.653 | 59679.04 | 38.3 | 40.9 | 20.0 | 0.00010 |
|  |  | Precentral | 2 | -6.069 | 756.64 | 19.4 | -9.3 | 55.8 | 0.00180 |

Table S7. Maximum values of significant clusters after cluster-wise correction for multiple comparison of mixed models comparing surface area in N-PS *vs* LA-PS individuals with 22q11DS using the *mri_surfcluster* function from FreeSurfer. Gender, scanner and intracranial volume were included as covariates in the analyses. Max = maximum -log10(p-value) found in the cluster. Size (mm^2^) = surface area of the cluster. Tal(X,Y,Z) = MNI coordinates of the maximum p-value. CWP = cluster-wise p-value.

| **Hemi-sphere** | **Model effect** | **Brain**  **Region** | **Cluster Number** | **Max** | **Size (mm^2^)** | **TalX** | **TalY** | **TalZ** | **CWP** |
| --- | --- | --- | --- | --- | --- | --- | --- | --- | --- |
| Left | Group effects | Middle temporal | 1 | -2.619 | 1019.63 | -58.9 | -52.8 | 1.3 | 0.00010 |
| Right | Group effects | Middle temporal | 1 | -1.820 | 824.62 | 64.4 | -34.3 | -8.3 | 0.00090 |
|  |  | Superior parietal | 2 | 2.365 | 539.00 | 21.3 | -58.7 | 49.7 | 0.01920 |
|  |  | Inferior parietal | 3 | -2.649 | 510.76 | 41.4 | -72.3 | 12.1 | 0.02630 |

## Supplementary Figures

Figure S1. Scan distribution for the exploratory analysis comparing three subgroups of patients with 22q11DS: a group without psychotic symptoms (N-PS, N=47), a group of individuals with lifetime attenuated psychotic symptoms who do not develop psychosis (LA-PS no psychosis, N=49), and a group of individuals with lifetime attenuated psychotic symptoms who develop psychosis (LA-PS with psychosis, N=12). The total sample consisted of 108 patients with 22q11DS aged 6-28 years who contributed between 1-5 scans, resulting in a total of 244 scans (N=98 N-PS; N=114 LA-PS no psychosis; N = 32 LA-PS with psychosis).

Figure S2. Scan distribution across the entire sample, colored by scanner. T1-weighted MRI anatomical brain scans were acquired using three different scanners: a 1.5T Philips Intera scanner (158 scans), a 3T Siemens Trio scanner (300 scans) and a 3T Siemens Prisma scanner (178 scans). The proportion of scans acquired with each scanner did not differ between 22q11DS and controls (p=0.07) or LA-PS and N-PS groups (p=0.83).

Figure S3. Scan distribution for each diagnostic group. Scanners were used across the developmental period and follow a similar distribution in 22q11DS and controls.


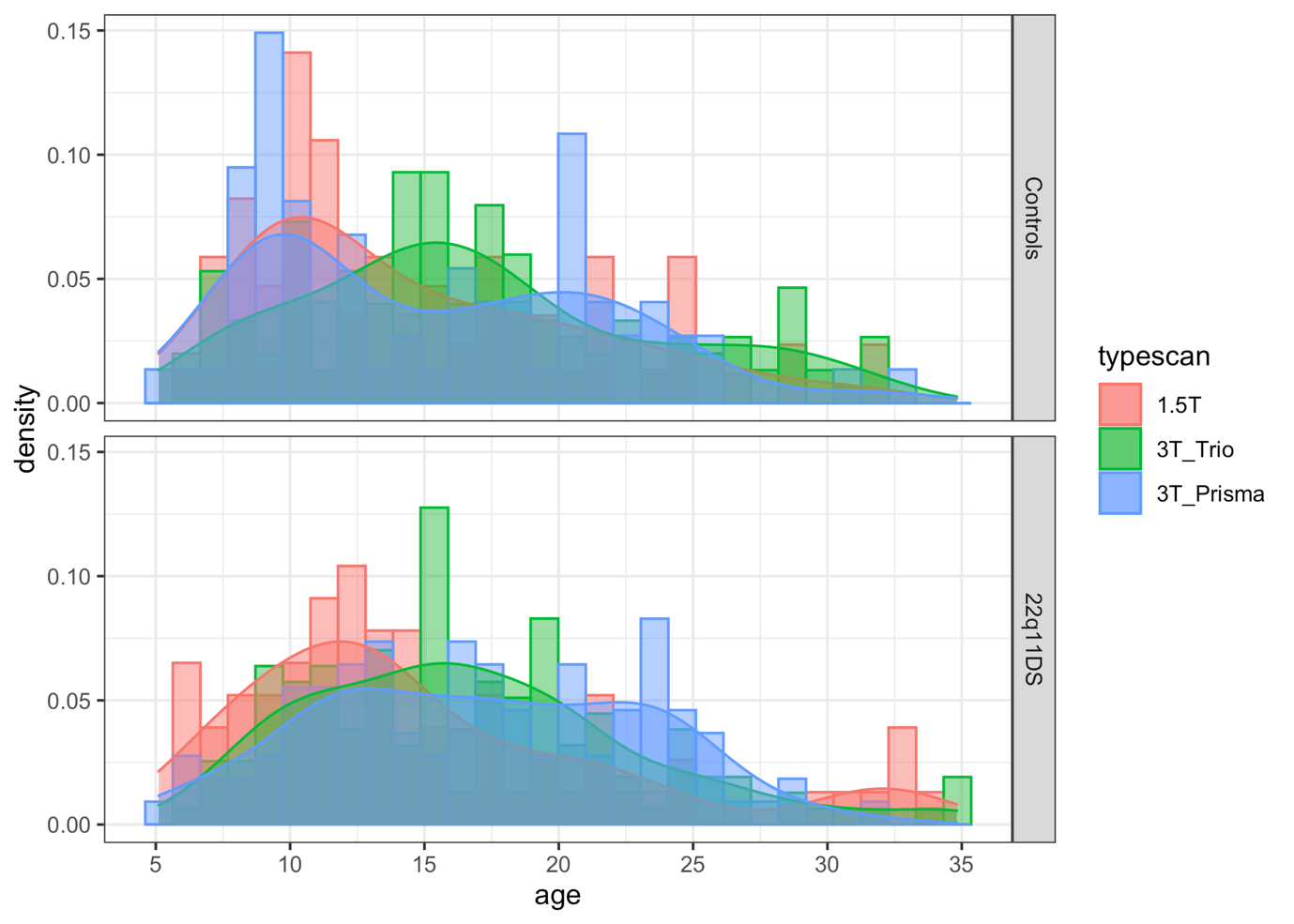


Figure S4. Relationship between a) total Intracranial Volume (ICV) and mean cortical thickness (averaged across both hemispheres); b) total Intracranial Volume (ICV) and total surface area (across hemispheres). Cortical thickness and ICV do not show any association; cortical surface area shows a clear linear association with ICV.


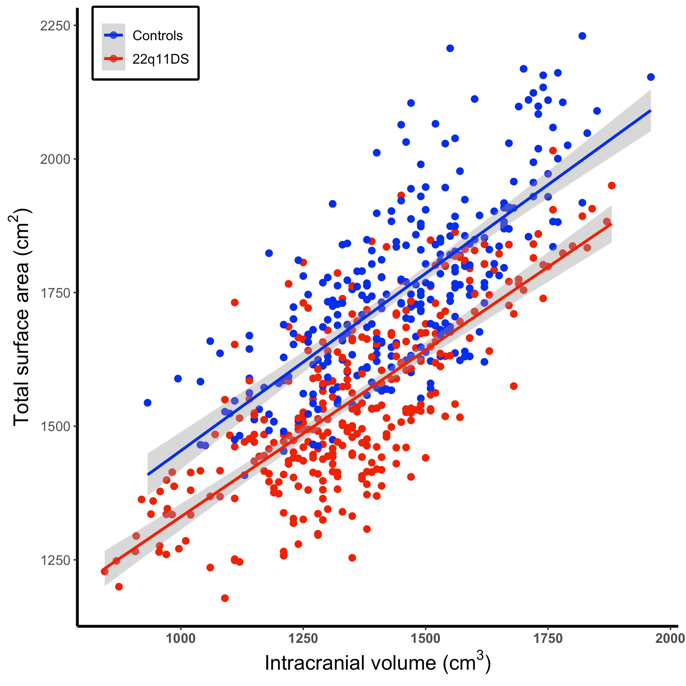

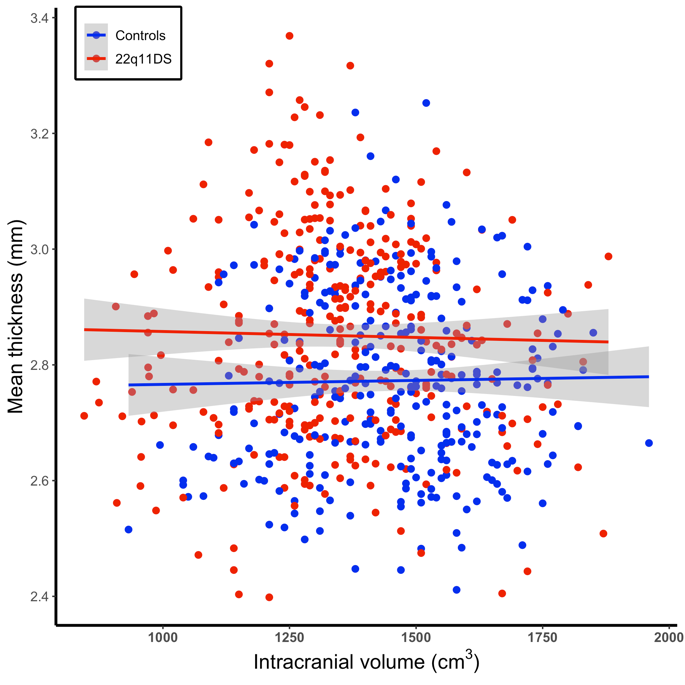


A

B

Figure S5. Significant intercept differences in cortical thickness between 22q11DS and controls after cluster-wise correction for multiple comparisons. In the central map, cold colors reflect increased cortical thickness and warm colors indicate reduced cortical thickness in 22q11DS compared to controls. The brain is shown in lateral, medial and inferior views. Individuals with 22q11DS show widespread increases in cortical thickness, with focal reductions in the superior temporal gyrus and in the posterior cingulate cortex. The upper central map depicts model orders fitted at each vertex, with dark red indicating constant models, orange corresponding to linear models and yellow indicating quadratic models.

Figure S6. Significant shape differences in cortical thickness between 22q11DS and controls after cluster-wise correction for multiple comparisons. In the central map, warm colors reflect the degree of significance of shape differences found at each vertex. The brain is shown in lateral, medial and inferior views. Individuals with 22q11DS show increased cortical thickness during childhood followed by accelerated thinning during adolescence, most prominently in fronto-temporal regions. The upper central map depicts model orders fitted at each vertex, with dark red indicating constant models, orange corresponding to linear models and yellow indicating quadratic models.

Figure S7. Time course displaying A) differences in cortical thickness and B) differences in the annual rate of cortical thinning between 22q11DS and controls from 5-35 years, in the right hemisphere. Cold colors reflect increased cortical thickness (A) or lower thinning rates (B) in 22q11DS compared to controls; warm colors indicate reduced cortical thickness (A) or higher thinning rates (B) in 22q11DS compared to controls. CT is increased throughout the cortex in 22q11DS compared to controls. CT differences tended to lessen during adolescence, although most differences remained evident throughout the brain in adulthood. Focal reductions in the posterior cingulate and superior temporal gyrus also remain present throughout development. Individuals with 22q11DS further show accelerated rates of thinning, most markedly in fronto-temporal regions. Interestingly, thinning rates become particularly exacerbated in certain regions when entering adulthood (e.g., the right insula and lateral occipital gyrus), reflecting a continued thinning process in individuals with 22q11DS. Videos displaying the evolution of cortical thickness and thinning rate differences between 22q11DS and controls over time and in the entire brain are available in the Supplementary Materials (Supplementary Video 1 for cortical thickness differences; Supplementary Video 2 for thinning rate differences).

Figure S8. Time course displaying A) differences in cortical thickness and B) differences in the annual rate of cortical thinning between LA-PS and N-PS subgroups of individuals with 22q11DS from 6-28 years, in the left hemisphere. Cold colors reflect increased cortical thickness (A) or lower thinning rates (B) in LA-PS compared to N-PS participants; warm colors indicate reduced cortical thickness (A) or higher thinning rates (B) in LA-PS compared to N-PS individuals. Patients with positive psychotic symptoms show overall reduced CT in several frontal, temporal and parietal regions. No differences in thinning rates are observed in the left hemisphere. Videos displaying the evolution of cortical thickness and thinning rate differences between LA-PS and N-PS groups over time and in the entire brain are available in the Supplementary Materials (Supplementary Video 3 for cortical thickness differences; Supplementary Video 4 for thinning rate differences).

Figure S9. Time course displaying A) differences in cortical thickness and B) differences in the annual rate of cortical thinning between LA-PS and N-PS subgroups of individuals with 22q11DS from 6-28 years, in the right hemisphere. Cold colors reflect increased cortical thickness (A) or lower thinning rates (B) in LA-PS compared to N-PS participants; warm colors indicate reduced cortical thickness (A) or higher thinning rates (B) in LA-PS compared to N-PS participants. While individuals with positive psychotic symptoms show initially increased CT in the right STG during childhood compared to individuals without psychotic symptoms, the increases progressively disappear until showing reduced CT. Thinning rates in the right STG are steeper in individuals with psychotic symptoms and remain constant throughout development. Videos displaying the evolution of cortical thickness and thinning rate differences between LA-PS and N-PS groups over time and in the entire brain are available in the Supplementary Materials (Supplementary Video 3 for cortical thickness differences; Supplementary Video 4 for thinning rate differences).

Figure S10. Brain maps of the exploratory analysis comparing three subgroups of individuals with 22q11DS: N-PS individuals, LA-PS individuals without overt psychosis, and LA-PS individuals with overt psychosis. In the upper left map, cold colors reflect increased cortical thickness and warm colors indicate reduced cortical thickness in LA-PS compared to N-PS individuals. The brain is shown in lateral, medial and inferior views. A significant group effect was found in the right superior frontal gyrus, where LA-PS patients with psychosis show markedly thinner cortex. In the lower left map, warm colors reflect the degree of significance of shape differences found at each vertex. The brain is shown in lateral, medial and inferior views. Significant interaction effects were found in the left lateral occipital gyrus and right STG. In these regions, LA-PS patients with psychosis show more exacerbated thinning compared to LA-PS patients without psychosis and N-PS patients. The upper right map represents model orders fitted at each vertex, with dark red indicating constant models and orange corresponding to linear models. Results should be considered as preliminary, due to the small sample size in the group of individuals with overt psychosis.

Figure S11. Significant intercept differences in surface area between 22q11DS and controls after cluster-wise correction for multiple comparisons. In the central map, cold colors reflect increased surface area and warm colors indicate reduced surface area in 22q11DS compared to controls. The brain is shown in lateral, medial and inferior views. Individuals with 22q11DS show a constant decrease in surface area in most cortical areas throughout development, except for precentral areas showing increased surface area. The upper central map depicts model orders fitted at each vertex, with dark red indicating constant models, orange corresponding to linear models and yellow indicating quadratic models. A majority of constant model orders were fitted, indicating no change in surface area during the included developmental period.


Figure S12. Significant shape differences in surface area between 22q11DS and controls after cluster-wise correction for multiple comparisons. In the central map, warm colors reflect the degree of significance of shape differences found at each vertex. The brain is shown in lateral, medial and superior views. While the left superior parietal gyrus undergoes a decrease in surface area during development, surface area levels remain constant in individuals with 22q11DS. The upper map represents model orders fitted at each vertex, with dark red indicating constant models, orange corresponding to linear models and yellow indicating quadratic models. A majority of constant model orders were fitted, indicating no change in surface area during the included developmental period.

Figure S13. Brain maps showing regions with significant intercept effects when comparing surface area in N-PS and LA-PS groups within 22q11DS. In the central brain map, cold colors reflect increased surface area and warm colors indicate reduced surface area in LA-PS compared to N-PS individuals. The brain is shown in lateral, medial and inferior views. Significant group differences were found in bilaterally in the middle temporal gyrus and in the right inferior parietal gyrus, where individuals with positive psychotic symptoms showed increased surface area compared to individuals without psychotic symptoms. By contrast, surface area was reduced in the right superior parietal gyrus in patients with positive psychotic symptoms. No group by age interaction effects were found, indicating that surface area did not show diverging developmental trajectories in individuals with positive psychotic symptoms. The upper map represents model orders fitted at each vertex, with dark red indicating constant models, orange corresponding to linear models and yellow corresponding to quadratic models.

## References

1. Schaer M, Debbané M, Bach Cuadra M, Ottet M-C, Glaser B, Thiran J-P, et al. Deviant trajectories of cortical maturation in 22q11.2 deletion syndrome (22q11DS): A cross-sectional and longitudinal study. Schizophr Res. 2009 Dec;115(2–3):182–90.

2. Schneider M, Schaer M, Mutlu AK, Menghetti S, Glaser B, Debbané M, et al. Clinical and cognitive risk factors for psychotic symptoms in 22q11.2 deletion syndrome: a transversal and longitudinal approach. Eur Child Adolesc Psychiatry. 2014 Jun 1;23(6):425–36.

3. Swillen A, McDonald‐McGinn D. Developmental trajectories in 22q11.2 deletion syndrome. Am J Med Genet C Semin Med Genet. 2015 Jun 1;169(2):172–81.

4. Wechsler D. Manual for the Wechsler intelligence scale for children-(WISC-III). San Antonio TX Psychol Corp. 1991;

5. Wechsler D. Wechsler adult intelligence scale-III. San Antonio, TX: The Psychological Corporation; 1997.

6. Wechsler D. Wechsler Adult Intelligence Scale–Fourth Edition (WAIS–IV). San Antonio, TX: The Psychological Corporation; 2008.

7. Wechsler D. WISC-IV: Wechsler Intelligence Scale for children, integrated: Technical and interpretive manual. Harcourt Brace and Company; 2004.

8. Reich W. Diagnostic Interview for Children and Adolescents (DICA). J Am Acad Child Adolesc Psychiatry. 2000 Jan 1;39(1):59–66.

9. Kaufman J, Birmaher B, Brent D, Rao U, Flynn C, Moreci P, et al. Schedule for Affective Disorders and Schizophrenia for School-Age Children-Present and Lifetime Version (K-SADS-PL): Initial Reliability and Validity Data. J Am Acad Child Adolesc Psychiatry. 1997 Jul 1;36(7):980–8.

10. First MB, Spitzer RL, Gibbon M, Williams JB. Structured clinical interview for DSM-IV clinical version (SCID-I/CV). Washington DC: American Psychiatric Press; 1997.

11. Mancini V, Sandini C, Padula MC, Zöller D, Schneider M, Schaer M, et al. Positive psychotic symptoms are associated with divergent developmental trajectories of hippocampal volume during late adolescence in patients with 22q11DS. Mol Psychiatry [Internet]. 2019 Jun 4 [cited 2019 Jul 4]; Available from: http://www.nature.com/articles/s41380-019-0443-z

12. Sun D, Ching CRK, Lin A, Forsyth JK, Kushan L, Vajdi A, et al. Large-scale mapping of cortical alterations in 22q11.2 deletion syndrome: Convergence with idiopathic psychosis and effects of deletion size. Mol Psychiatry. 2018 Jun 13;1–13.

13. Skåtun KC, Kaufmann T, Doan NT, Alnæs D, Córdova-Palomera A, Jönsson EG, et al. Consistent Functional Connectivity Alterations in Schizophrenia Spectrum Disorder: A Multisite Study. Schizophr Bull. 2017 Jul 1;43(4):914–24.

14. Han X, Jovicich J, Salat D, van der Kouwe A, Quinn B, Czanner S, et al. Reliability of MRI-derived measurements of human cerebral cortical thickness: The effects of field strength, scanner upgrade and manufacturer. NeuroImage. 2006 Aug 1;32(1):180–94.

15. Mutlu AK, Schneider M, Debbané M, Badoud D, Eliez S, Schaer M. Sex differences in thickness, and folding developments throughout the cortex. NeuroImage. 2013 Nov 15;82:200–7.

16. Dale AM, Fischl B, Sereno MI. Cortical Surface-Based Analysis: I. Segmentation and Surface Reconstruction. NeuroImage. 1999 Feb 1;9(2):179–94.

17. Fischl B, Dale AM. Measuring the thickness of the human cerebral cortex from magnetic resonance images. Proc Natl Acad Sci. 2000 Sep 26;97(20):11050–5.

18. Fischl B, Sereno MI, Tootell RBH, Dale AM. High-resolution intersubject averaging and a coordinate system for the cortical surface. Hum Brain Mapp. 1999;8(4):272–84.

19. Fischl B, Sereno MI, Dale AM. Cortical surface-based analysis: II: inflation, flattening, and a surface-based coordinate system. Neuroimage. 1999;9(2):195–207.

20. Jalbrzikowski M, Jonas R, Senturk D, Patel A, Chow C, Green MF, et al. Structural abnormalities in cortical volume, thickness, and surface area in 22q11.2 microdeletion syndrome: Relationship with psychotic symptoms. NeuroImage Clin. 2013 Jan 1;3:405–15.

21. Gudbrandsen M, Daly E, Murphy CM, Blackmore CE, Rogdaki M, Mann C, et al. Brain morphometry in 22q11.2 deletion syndrome: an exploration of differences in cortical thickness, surface area, and their contribution to cortical volume. Sci Rep. 2020 Nov 2;10(1):18845.

22. Schmitt JE, Vandekar S, Yi J, Calkins ME, Ruparel K, Roalf DR, et al. Aberrant Cortical Morphometry in the 22q11.2 Deletion Syndrome. Biol Psychiatry. 2015 Jul 15;78(2):135–43.

23. Tamnes CK, Herting MM, Goddings A-L, Meuwese R, Blakemore S-J, Dahl RE, et al. Development of the Cerebral Cortex across Adolescence: A Multisample Study of Inter-Related Longitudinal Changes in Cortical Volume, Surface Area, and Thickness. J Neurosci. 2017 Mar 22;37(12):3402–12.

24. Vijayakumar N, Allen NB, Youssef G, Dennison M, Yücel M, Simmons JG, et al. Brain development during adolescence: A mixed-longitudinal investigation of cortical thickness, surface area, and volume. Hum Brain Mapp. 2016;37(6):2027–38.

25. Wierenga LM, Langen M, Oranje B, Durston S. Unique developmental trajectories of cortical thickness and surface area. NeuroImage. 2014 Feb 15;87:120–6.

26. Raznahan A, Shaw P, Lalonde F, Stockman M, Wallace GL, Greenstein D, et al. How Does Your Cortex Grow? J Neurosci. 2011 May 11;31(19):7174–7.

27. Ducharme S, Albaugh MD, Nguyen T-V, Hudziak JJ, Mateos-Pérez JM, Labbe A, et al. Trajectories of cortical surface area and cortical volume maturation in normal brain development. Data Brief. 2015 Dec 1;5:929–38.

28. Rakic P. A small step for the cell, a giant leap for mankind: a hypothesis of neocortical expansion during evolution. Trends Neurosci. 1995 Sep 1;18(9):383–8.

29. Rakic P. Evolution of the neocortex: a perspective from developmental biology. Nat Rev Neurosci. 2009 Oct;10(10):724–35.

30. Forsyth JK, Mennigen E, Lin A, Sun D, Vajdi A, Kushan-Wells L, et al. Prioritizing Genetic Contributors to Cortical Alterations in 22q11.2 Deletion Syndrome Using Imaging Transcriptomics. Cereb Cortex. 2021 Feb 26;bhab008.

31. Ramanathan S, Mattiaccio LM, Coman IL, Botti J-AC, Fremont W, Faraone SV, et al. Longitudinal trajectories of cortical thickness as a biomarker for psychosis in individuals with 22q11.2 deletion syndrome. Schizophr Res. 2017 Oct 1;188:35–41.

32. Rimol LM, Nesvåg R, Hagler DJ, Bergmann Ø, Fennema-Notestine C, Hartberg CB, et al. Cortical Volume, Surface Area, and Thickness in Schizophrenia and Bipolar Disorder. Biol Psychiatry. 2012 Mar 15;71(6):552–60.

33. Palaniyappan L, Mallikarjun P, Joseph V, White TP, Liddle PF. Regional contraction of brain surface area involves three large-scale networks in schizophrenia. Schizophr Res. 2011 Jul 1;129(2):163–8.

34. van Erp TGM, Walton E, Hibar DP, Schmaal L, Jiang W, Glahn DC, et al. Cortical Brain Abnormalities in 4474 Individuals With Schizophrenia and 5098 Control Subjects via the Enhancing Neuro Imaging Genetics Through Meta Analysis (ENIGMA) Consortium. Biol Psychiatry. 2018 Nov 1;84(9):644–54.

35. Padula MC, Schaer M, Armando M, Sandini C, Zöller D, Scariati E, et al. Cortical morphology development in patients with 22q11.2 deletion syndrome at ultra-high risk of psychosis. Psychol Med. 2018 Jan 17;1–9.

36. Bois C, Ronan L, Levita L, Whalley HC, Giles S, McIntosh AM, et al. Cortical Surface Area Differentiates Familial High Risk Individuals Who Go on to Develop Schizophrenia. Biol Psychiatry. 2015 Sep 15;78(6):413–20.
